# A collection of 3D geomodels of the Los Humeros and Acoculco geothermal systems (Mexico)

**DOI:** 10.1038/s41597-022-01327-0

**Published:** 2022-06-08

**Authors:** Philippe Calcagno, Eugenio Trumpy, Luis Carlos Gutiérrez-Negrín, Domenico Liotta

**Affiliations:** 1 Geothermal Division, Bureau de Recherches Géologiques et Minières (BRGM), Orléans, France; 2 Institute of Geosciences and Earth Resources, Consiglio Nazionale delle Ricerche (CNR), Pisa, Italy; 3 Geoconsul, SA de CV, Morelia, Mexico; 4 Department of Earth and Geo-Environmental Sciences, Aldo Moro University, Bari, Italy

**Keywords:** Geophysics, Structural geology, Geochemistry, Volcanology, Hydrogeology

## Abstract

This paper aims at sharing 3D geological models that were constructed at different scales in two Mexican geothermal areas as part of the European-Mexican GEMex project. The project was devoted to investigate superhot resources in Los Humeros and enhanced geothermal systems in Acoculco, both areas located in eastern Mexico. To build confidence in the resultant datasets and to potentially inform the development of models in similar contexts, the methodology is also described. The models integrate the main geological and geothermal features of the study areas and served as a framework for subsequent calculations and simulations. Preliminary models were based on data available at the beginning of the project, and were updated several times as new geological, geochemical, and geophysical field-data were obtained. The construction of the geomodels was performed in a collaborative and interdisciplinary way, using an existing software, and ultimately enabled a consensus interpretation and representation to be reached by the several disciplinary experts involved.

## Abstract (summary)

**Table:** 

Measurement(s)	geological features
Technology Type(s)	3D geomodelling integration
Sample Characteristic - Location	Los Humeros geothermal field (MX) • Acoculco geothermal field (MX)

## Background & Summary

The purpose of this paper is to make the three-dimensional (3D) models of subsurface structures (hereafter geomodels) constructed during the GEMex project^1^ accessible and reusable for further studies. To this end, they are shared in various formats. The geomodels provide a framework for simulating underground processes, such as groundwater flow, and can be reused for the development of the Los Humeros and Acoculco geothermal fields. They could also be further refined, improved or adapted for other purposes located in the area. In addition, this paper aims at providing an example of a methodology that could be reused in future studies to integrate multi-scale information and data from different disciplines.

In underground exploration activity, merging of data deriving from different Earth Sciences disciplines is a skilled task which is often achieved via a geomodel. Therefore, the methodology to integrate data is the key factor for a successful, reasonable and scientifically acceptable result. A geomodel summarizes geological knowledge based on various types of input data, and it is a prerequisite for further simulations based on geological geometry and parameters. Lifetimes of a geological models are often limited to the studies they were constructed for. On top of that, such models are rarely made freely available for potential reuse. However, such geomodels, if well constructed, are a precious source of information that can be both further refined when new data become available, and reused for other studies in the area.

Sound knowledge of subsurface structures and geological formations is critically dependent on the capability for 3D consistent interpretation and visualisation^2–4^. In the past decade, significant advancements have been made in the development of 3D mapping, visualisation and modelling for geothermal purposes^5,6^. Interdisciplinary integration is a key factor for a robust and coherent knowledge of the investigated area. Usually, integration consists in combining data from various disciplines, with the work being done by a single person. That makes collaborative scientific reasoning difficult, and often overlooks crucial questions such as: “Is the knowledge acquired during a field campaign fully contained in the input data?” or “How is the geologist’s experience used when interpreting geophysical data?”

**Fig. 1:**
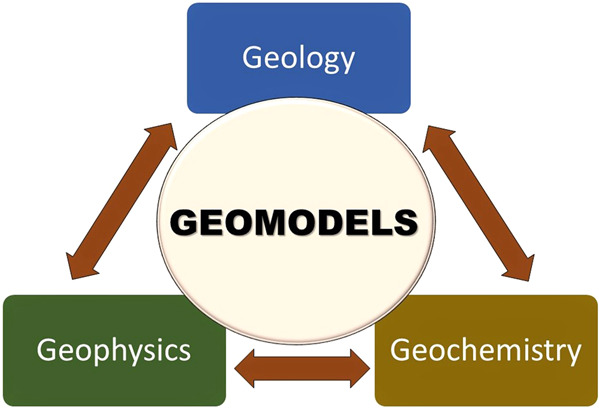
Conceptual illustration showing the interdisciplinary collaboration via 3D geomodelling^7^. The model is a co-directed, mutual, shared, and robust representation of the targeted subsurface region. The principle is illustrated on the schema with the main complementary fields used in the modelling process.

The work presented in this paper goes a step forward by developing the contributions from the various disciplines together in a single 3D integration platform (Fig. 1), beyond the data themselves, with the direct participation of several specialists in most of the stages of the project^7^. In this way, the geomodels presented in this study are not the final result of a sequential and barely connected integration, but are a central tool of a cooperative interpretation process, leading to mutually accepted results.

**Fig. 2:**
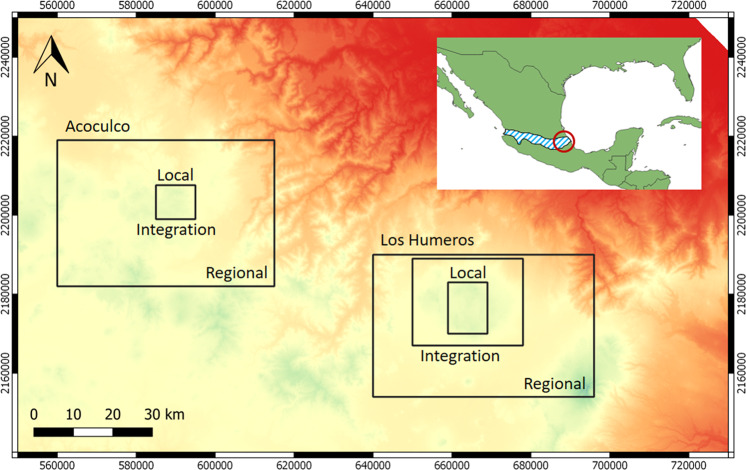
Locations and extents of the study areas, eastern of the Trans-Mexican Volcanic Belt (dashed blue area in the insert). The black rectangles present three areas for Los Humeros geomodels: (i) regional, (ii) local, and (iii) integration, and two areas for Acoculco geomodels: (i) regional and (ii) local/integration. Area locations are shown on the 90 m Digital Elevation Model SRTM. The coordinate system is WGS84/UTM zone 14 N.

The GEMex project was focused on geothermal energy resources^8^, and sought to combine Mexican practical expertise with European technical capabilities. It was a project of the EU’s Horizon 2020 research and innovation programme that ran from 2016 to 2020 and involved the participation of 33 European and Mexican partners^9^. More information about the project structure and contents can be found at www.gemex-h2020.eu. Two sites were dedicated to GEMex: Los Humeros and Acoculco, both located in eastern Mexico (Fig. 2). They are situated within the Trans-Mexican Volcanic Belt (TMVB), which is a continental volcanic arc crossing central Mexico. Volcanic and seismic activities have occurred along the TMVB since about 16 Ma ago^10^, with several currently active volcanoes (e.g. Popocatépetl, Volcán de Colima). Three of the five conventional geothermal fields under exploitation in Mexico are located in the TMVB.

An important ambition was to develop coherent, comprehensive, and reliable 3D geomodels to: (a) gather and geolocate data and information from various disciplines, (b) serve as reference for further computations and simulations, (c) help to understand the geothermal systems. The existing information related to the study areas was compiled in an integrated 3D model framework, which was continuously updated as new data and information came in, thus providing a platform to integrate the various project results across different scales of investigation (Fig. 2).

The geomodels presented in this paper synthetize the investigations made in Los Humeros and Acoculco at different scales (Fig. 2) and update levels. The various geomodel versions were made available regularly to project partners and were used as a quantitative geometrical basis for additional works, such as compiling rock properties^11^, calculating seismic properties^12^, computing earthquake tomography^13^, evaluating thermal behaviour and geothermal potential^14^, modelling fluid reinjection^15^, and interpreting the volcanic and geothermal systems^16–18^.

## Geological and Geothermal Frameworks

Los Humeros, located approximately 2800 m above sea level, is one of the five geothermal fields currently operating in Mexico. Their combined power capacity is about 1000 MW (megawatts). The field has been developed inside two nested calderas named Los Humeros and Los Potreros. The former one is 18–20 km in diameter and was formed 165 ka ago, while the latter caldera is 5–8 km in diameter and was formed around 70 ka^19^ ago.

The public utility Comisión Federal de Electricity (CFE) holds the exploitation concession of Los Humeros, and started its operations in the 1990s. The current installed capacity is 119.8 MW, and the running or operational capacity is 94.8 MW, with a power generation of around 500 gigawatt-hours annually^20^. This represents approximately the 10% of the geothermal-electric generation of Mexico, and about 0.2% of the total electric generation in the country.

The exploited fluids are of conventional hydrothermal type, hosted in the andesites that mainly form the pre-caldera lithological group. The wells produce a mixing with more than 85–90% of high-temperature steam and 10–15% liquid phase, and only one well (H-1 and its successors H-1D and H-49) produces mainly liquid phase of sodium-chloride and bicarbonate-sulfate composition^20^. The highest temperature recorded in the wells is almost 400 °C, thus the field was chosen for developing the superhot part of the GEMex Project, which sought to define the probable location of the superhot fluids hosting rocks at depth. As interpreted while constructing the geomodels, these superhot fluids are probably contained in the upper portions of the underlying basement, and in the deepest parts of the pre-caldera volcanic rocks. In all cases, rock-permeability is basically secondary, due to faults and fractures belonging to the two main fault systems, roughly NW-and SW-trending, respectively.

The Acoculco geothermal zone is also located at the eastern portion of the TMVB, where NE-SW, NW-SE and minor E-W striking structural systems intersect each other within a regional extensional regime^21^. The basement rocks are made up of granitoids, Cretaceous limestone, local skarn, and marble overlain by basalts and Miocene pre-caldera domes and lavas between 13 and 3 Ma in age^16^. The Acoculco Caldera was formed 2.7 Ma ago and is an asymmetric caldera with sides of 16 and 18 km in length, and rhombohedral to sub-circular geometry. Since then, volcanic activity persisted up to around 60 ka^16,21,22^.

CFE holds the exploration permit in the area, and has drilled two exploratory wells: EAC-1 in 1995 and EAC-2 in 2008. The wells penetrate down to 2000 and 1900 m depth, and their maximum recorded temperatures are 307 °C and 264 °C, respectively. None of the wells produced fluids^23^, and so the zone was investigated with the aim of developing Enhanced/Engineered Geothermal System (EGS) technologies. Accordingly, the geothermal target is located in the basement, where the currently low permeability can be stimulated by hydro-fracturing. By considering fieldwork results^24^, we included in the Acoculco geomodels the most favourable geological structures suitable to be stimulated to increase permeability at depth, both to characterise their location and extent, and to allow future calculation based on their geometry.

## Methods

### Geomodelling approach

**Fig. 3:**
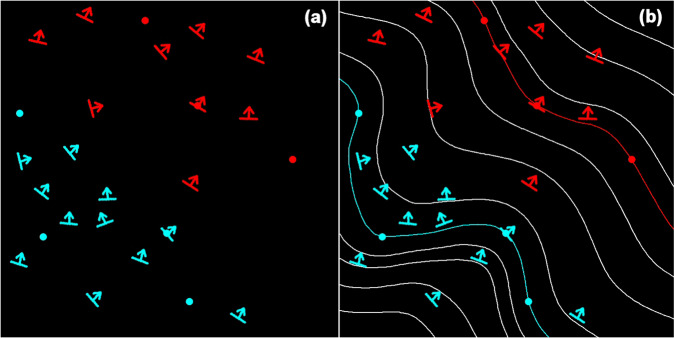
Interpolation method^25,26^ illustrated for two hypothetical geological formations, red and blue. (**a**) Input data for the interpolation: 3D points (location of geological interfaces) and 3D vectors (azimuth and dip of geological structures). (**b**) 3D potential field interpolation: The geological interfaces are modelled by isovalues of the potential field.

There are various approaches to construct 3D geomodels^2–4^. In this study, the interpolation of the input data was performed using a co-kriging geostatistical method, where 3D points located on the geological interfaces to be modelled and 3D vectors showing the dips of these geological interfaces are used at the same time^25^. 3D points and vectors are geological interfaces and dips respectively, either observed in the field or in boreholes, or interpreted by geologists. This method results in a 3D scalar potential field where isovalues represent geological interfaces (Fig. 3). A geological pile describes the chronological and topological relations between the various geological formations considered. It allows automatic management of the geological boundaries (gradual or erosional). The links between faults and formations are also described in the modelling process, to compute automatically how faults affect the formations. When faults interact with each other, they are combined in a fault network which describes their relations. This methodology^26^ is implemented in the commercial GeoModeller package (see “Code availability” section).

As described above, the geological and geothermal settings of Los Humeros and Acoculco are quite different and this was taken into account in the construction of the geological models. However, particular attention was paid to ensure a coherent geological interpretation of both areas, especially when similar geological objects were modelled in both sites.

### Los Humeros

The Los Humeros geomodels were constructed at three scales corresponding to the needs of the investigation (Fig. 2):

- A **local** scale focussing on the Los Humeros geothermal exploitation.
- A **regional** scale corresponding the area covered by the geological map^27^.
- An **integration** scale mainly based on the extent of the geophysical surveys performed during the GEMex project.

The following sections present the input data and information, the adopted geological descriptions, and the detailed integration process that was followed to construct the geomodels.

#### Data and information

A wide range of data and information were used to set up the Los Humeros geomodels. Digital Elevation Model (DEM), geological map and sections, wells, analogue model, geochemistry information, and geophysical models were all used to constrain the successive versions of the geomodels.

Geological information from only sixteen wells was available for the preliminary study^28^. A total of 56 wells were used for the updated local and integrated geomodels. The new lithological and structural information provided by the 40 additional wells resulted in a more detailed local model that changed our perception of the upper geometry of the basement rocks and the relevance of some of the main faults.

Table 1 specifies the data used for the preliminary and updated geomodels, as well as the additional data for the integrated geomodel. References are provided for each data and the way in which they were incorporated and used for the modelling is described.

**Table 1:** List and sources of datasets used for the construction of the Los Humeros geomodels.

Geomodel version	Data	Use	Integration
Preliminary and updated geomodels	DEM (30 m horizontal resolution)^46^	To reproduce topographic surface	Imported as .tif
	Geological map^27^	To define the group of formations to be modelled - Compared with the resulting model (at surface)	Imported as .tif
	CFE structural map (autoCAD)	Las Víboras fault interpretation	Imported as .tif
	Geological cross-sections^30,31^	To define the geological structures at depth	imported as .tif
	Faults system network^24^,^CFE*confidentialdata*^	To define fractured zones	Imported as .tif
	Wells ^CFE confidential data^	Constrain geological groups at depth	Imported as .csv
Integrated geomodel	Analogue modelling^47,48^	Guide to interpret the shapes of the structures	Qualitative information
	Regional fault system network^49^	Main structures in the basement	Qualitative information
	Geochemistry and hydrology^50^	Guide for the regional faults interpretation	Qualitative information
	3D resistivity model^51,52^	Main fault systems - Used to integrate geophysical datasets in the cross-plotting	Imported as .csv
	Seismic structures^13^	Las Papas fault interpretation at depth	Qualitative information
	Active seismic lines^53^	To constrain the main fault surfaces	Imported as .jpg
	3D gravity model^54^	Regional faults location - Basement interpretation - Constrain the Antigua fault: range 0.2 to 0.713875 (max) - Used to integrate geophysical datasets in the cross-plotting	Imported as .csv
	Integrated geophysical model^55^	Imaging and validation of geological structures	Qualitative information
	Results from cross-plotting data integration^33^	Used as imported surfaces to check and refine the geomodel surfaces	Imported as .csv
	Thermal modelling^56,57^	Used to define the new intrusion shape, imported isotherm	Imported as .csv
	Volcanoes morphology and distribution	Regional faults traces interpretation (volcanoes alignment)	Imported as .tif

The table indicates the purpose the dataset was used for and the way it was integrated in the geomodel. In the last column, “Qualitative information” means that the information was not directly imported but was rather used for checking and evaluating the geomodel.

#### Geological descriptions

Two approaches were applied to describe the underground lithology in Los Humeros, and correlate it with the outcropping lithological units. In the first approach, the subsurface rocks were described as four groups to be considered at the regional and integration scales in the related geomodels. In the second one, the same subsurface rocks were divided in nine units to be considered at the local scale in the related geomodel.

**Fig. 4:**
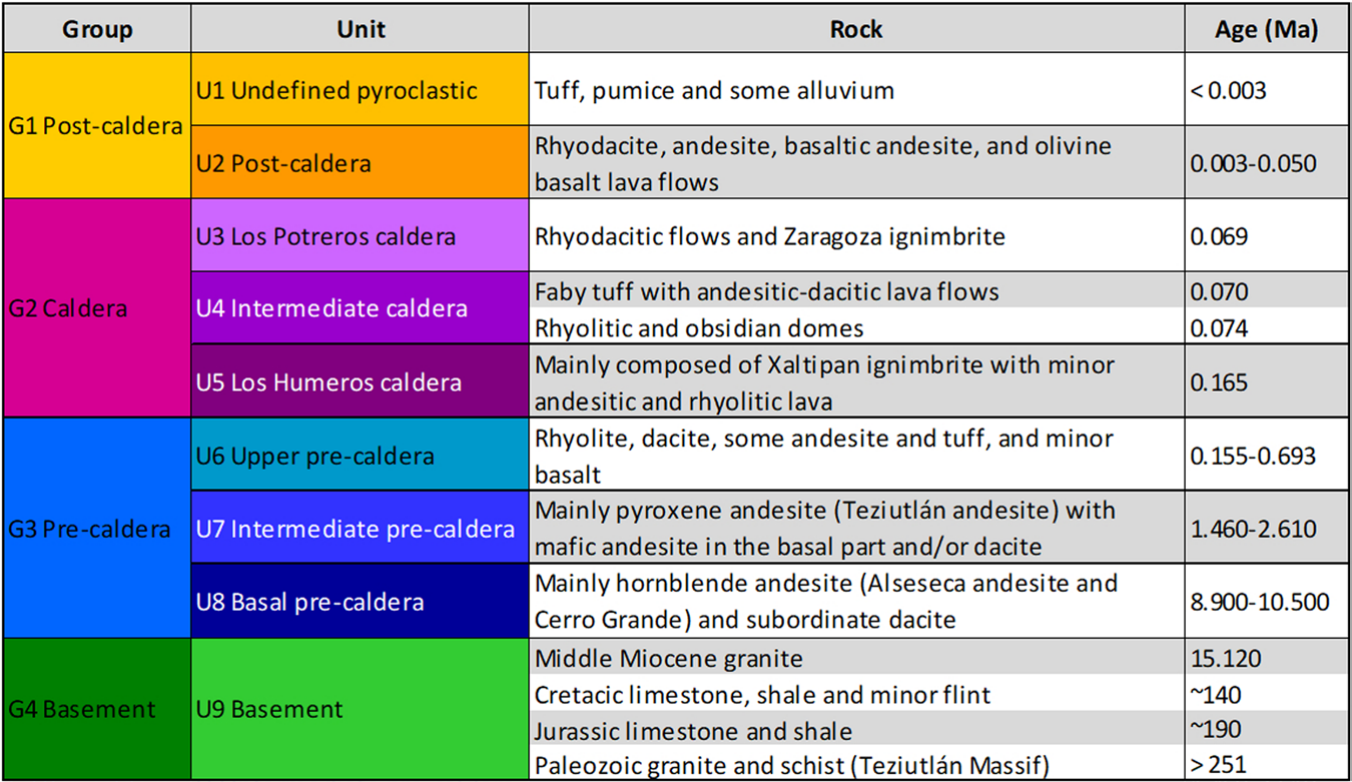
Geological description, 2017 version^28^.

The lithological description shown in Fig. 4 (2017 version) was used to construct the preliminary regional and local 3D geomodels^28^.

**Fig. 5:**
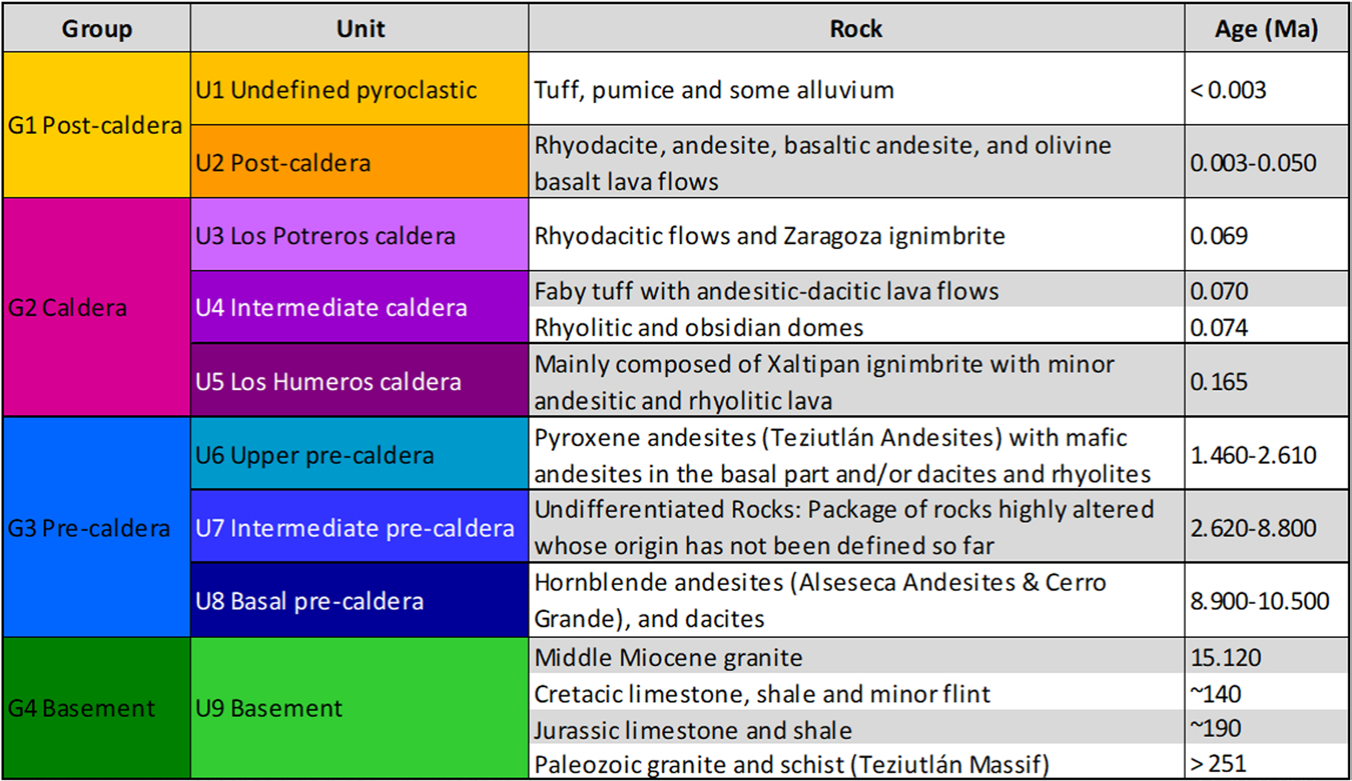
Geological description, 2018 version^29^. The three units of G3 are different from the 2017 version.

In 2018, the description in nine units was slightly modified. Upon the suggestion of the GEMex team and CFE’s geologists, it was decided to include a set of rocks of mainly pyroclastic origin within the Group G3. This new unit was identified by the CFE in the lithological columns of its wells as lithic, vitreous or crystalline tuffs (Tobas Líticas, Tobas Vítreas, Tobas Cristalinas). It is typically located between the upper pyroxene andesites (AP) and the lower hornblende andesites (AH), as shown in Fig. 5. Although this pyroclastic unit is not related to any outcropping unit and its origin remains uncertain, it has been included in the model because its petrophysical properties are in any case different from the andesitic rocks.

The geological description presented in Fig. 5 was used to construct the updated local 3D geomodel^29^.

#### Integration process

This section describes the steps we followed during the construction of the geomodels at the three different scales: local, regional, and integration (Fig. 2).

The Los Humeros geomodels were initiated at the outset of the GEMex project. Preliminary versions were constructed to give a coherent geological interpretation using the available information. They were then updated following the acquisition of new data in the field. A final version was produced that integrated geophysical information.

**Fig. 6:**
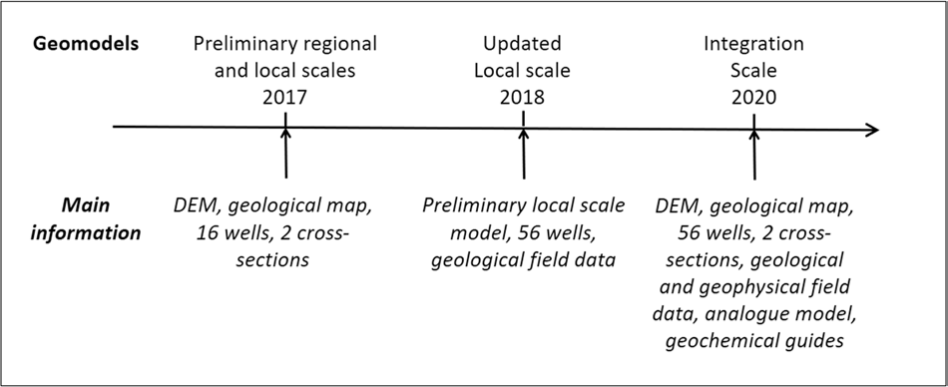
Los Humeros geomodels versions through time: preliminary^28^, updated^29^, and integrated. The main input information is listed for each of them.

Figure 6 presents the main steps of the Los Humeros geomodels that have been produced during the project.

#### Preliminary geomodels

The geological map^27^, two geological sections^30,31^, and the lithological columns of 16 wells provided by CFE were the main data sources used to set up the geomodels. In addition, CFE made available the geological descriptions of the considered wells. Considering the lack of information on their precise trajectories, the wells were assumed as vertical. The Digital Elevation Model (DEM) was provided by INEGI (Instituto Nacional de Estadística y Geografía), and had a 30 m horizontal resolution.

It was necessary to preselect the main faults to be modelled at the regional and local scales. The main faults are assumed to die out into the brittle ductile transition, supposedly located at four kilometers below ground level. (b.g.l.), considering the local temperature gradient.

For the modelling process, the geological formations were described as four groups and nine units, respectively at the regional and the local scales, using the 2017 version of the geological description (Fig. 4).

### Regional scale

The regional scale geomodel^28^ (56 km × 36 km × 12 km, i.e. down to 7 km below sea level) contains four geological groups: the basement, pre-caldera rocks, rocks from the caldera collapses, and post-caldera rocks (2017 version, Fig. 4). 16 faults were modelled at regional scale. The geological map^27^ and sections AA’^30^ and BB’^31^ were re-interpreted to be coherent with the four groups. The geological description of the wells made it possible to match all the information with the four groups selected for the modelling of the regional model.

**Fig. 7:**
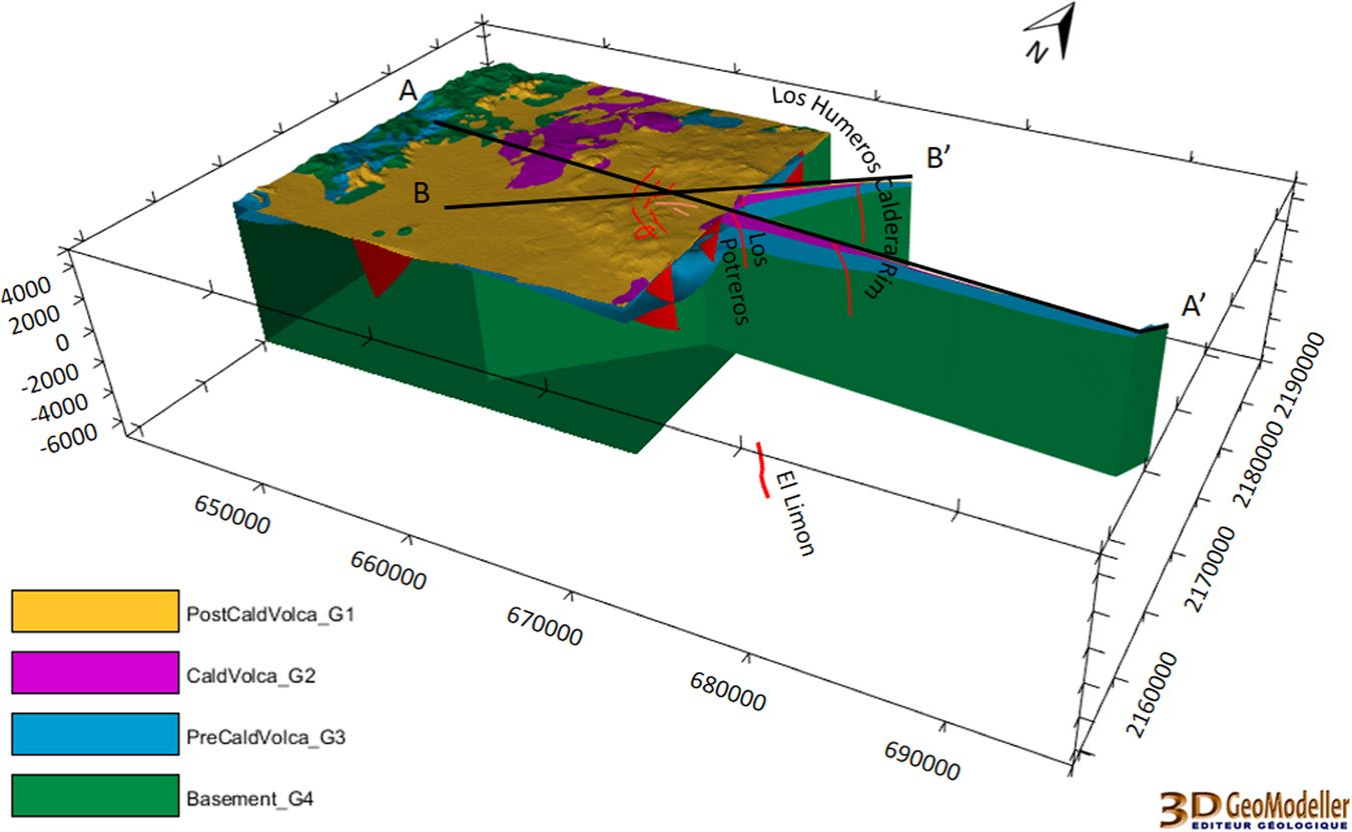
The Los Humeros regional geomodel of the four geological groups^28^ (see Fig. 4). Red lines display fault location. The coordinate system is WGS84/UTM zone 14 N.

The regional geomodel is presented in Fig. 7.

### Local scale

The local scale geomodel^28^ (9.5 km × 12.5 km × 12 km, i.e. down to 7 km below sea level) presents nine units (2017 version, Fig. 4). 18 faults were modelled at local scale. The geological map^27^, geological cross sections AA’^30^ and BB’^31^, and the 16 available wells have been re-interpreted accordingly.

**Fig. 8:**
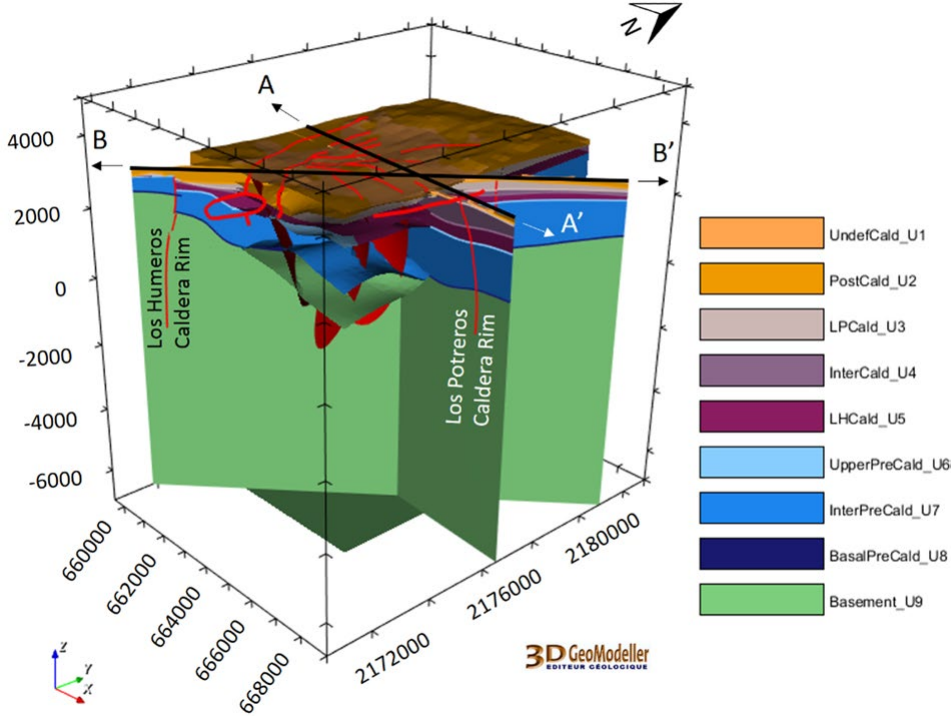
The Los Humeros local geomodel of the nine geological units^28^ (see Fig. 4). The coordinate system is WGS84/UTM zone 14 N.

The local geomodel is presented in Fig. 8.

#### Updated local scale

**Fig. 9:**
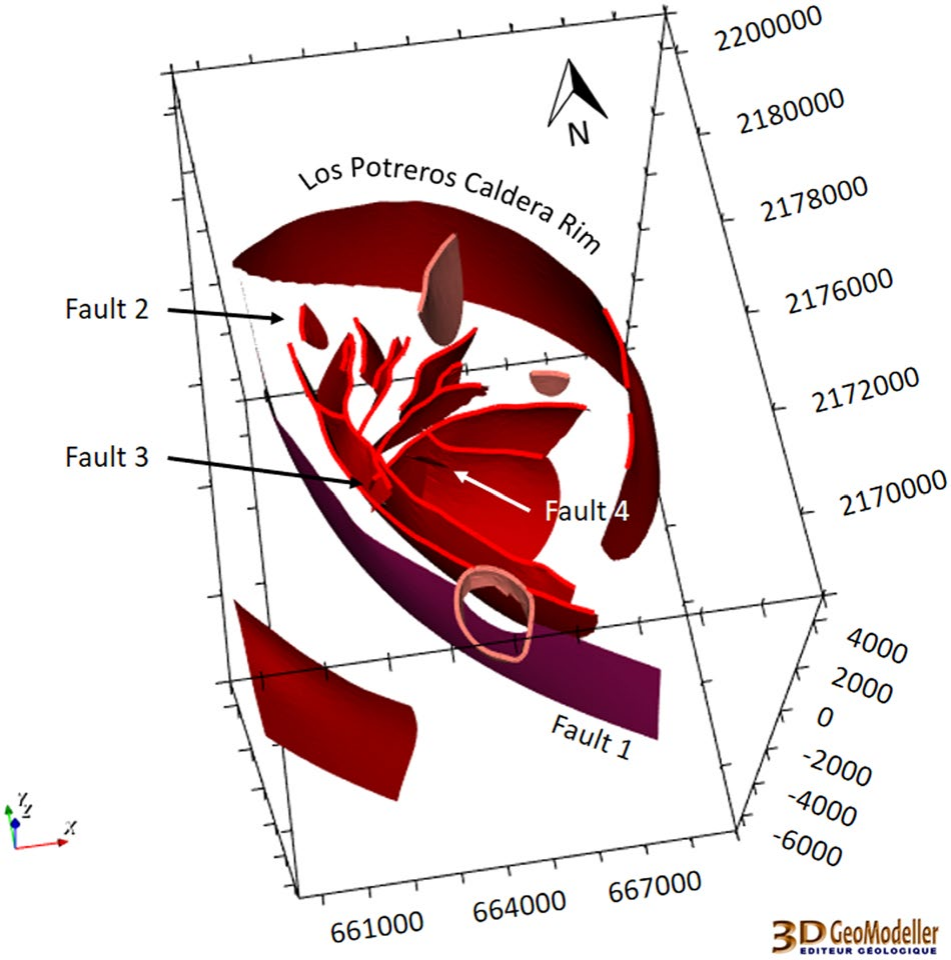
New fieldwork data led almost all of the faults in the local model of Los Humeros to be updated, as well as the addition of Faults 1 to 4^29^. The coordinate system is WGS84/UTM zone 14 N.

This version is an update of the local geomodel. It was improved by using new information regarding the faults and more wells provided by CFE. The update^29^ started with the refinement of the faults at local scale following new geological fieldwork^24^, as shown in Fig. 9. 21 faults were modelled in the updated local scale geomodel.

**Fig. 10:**
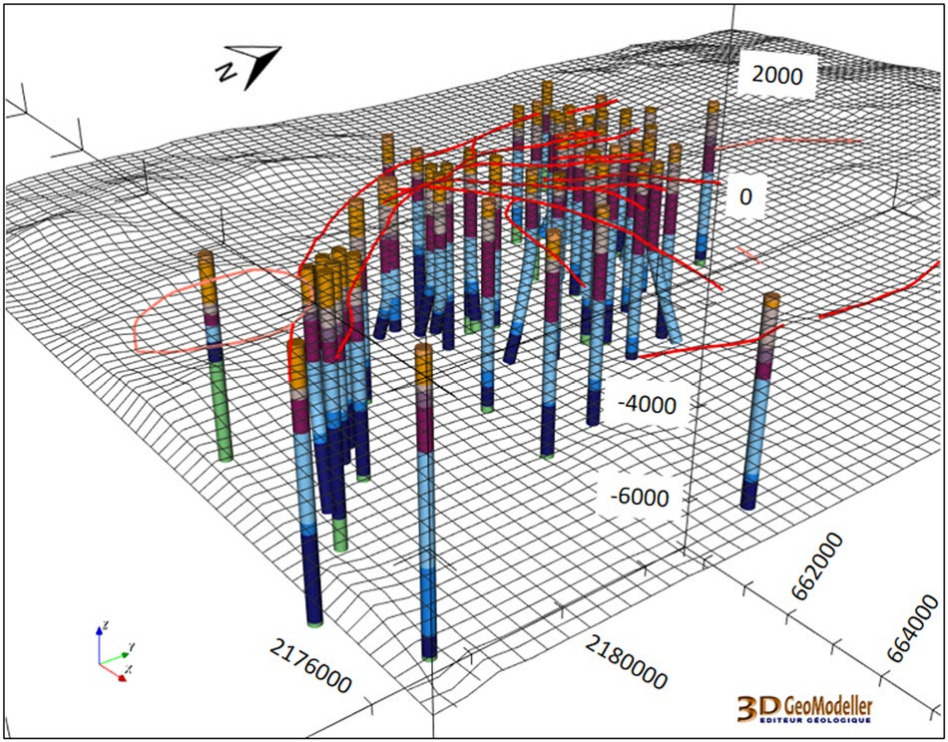
56 wells were used for the update of the Los Humeros local model (Updated local scale)^29^. Ten of them are deviated, i.e. non-vertical. They are described according to the 2018 geological description presented in Fig. 5. DEM is displayed as a grid including the fault network traces (see Fig. 9). View from SE.

In a second phase, the geological units were refined at local scale^29^. This step was mainly based on a new geological description of the nine units (2018 version, Fig. 5) and on 40 more wells from the CFE. A total of 56 wells were used to update the Los Humeros local area (Fig. 10). The wells were provided by CFE, including their trajectories if not vertical.

**Fig. 11:**
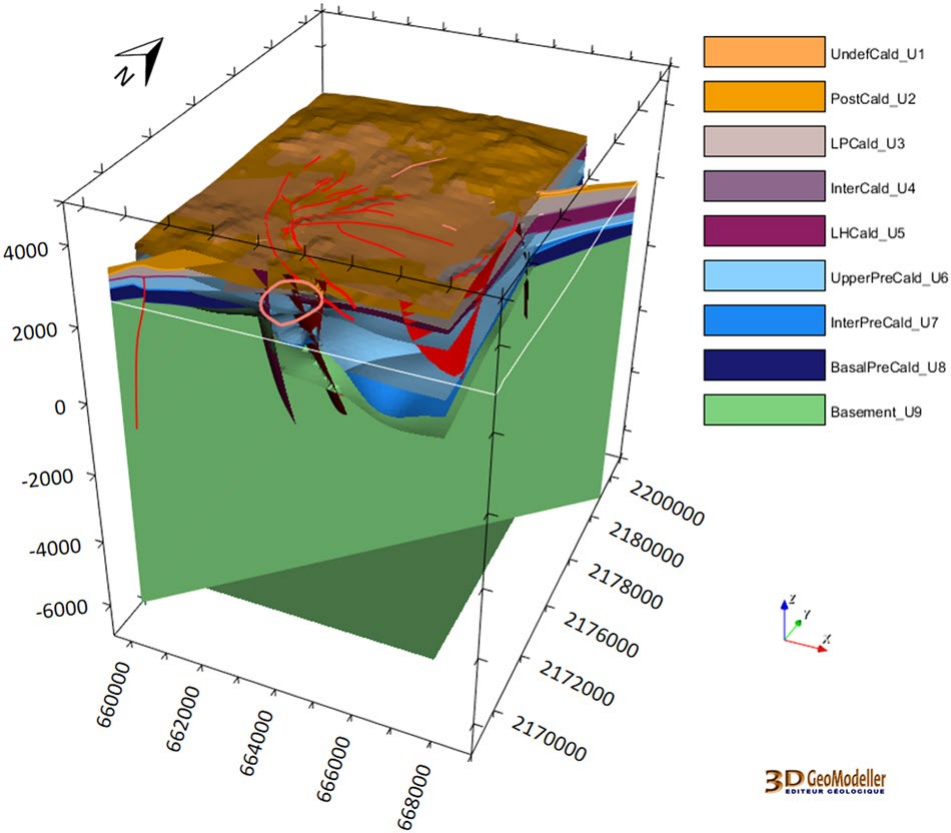
The 3D geomodel of Los Humeros updated at the local scale for the nine geological units^29^ (see Fig. 5). It includes the updated faults (Fig. 9) and 56 wells (Fig. 10). The coordinate system is WGS84/UTM zone 14 N.

The updated local-scale geomodel is presented in Fig. 11.

#### Integration scale

20 six faults were modelled at the integration scale.

The preliminary and updated geomodels of Los Humeros, which were constructed in the first part of GEMex, were mainly based on geological knowledge and data. In the second half of the project, inputs from other disciplines became available, such as analogue modelling (guide for shaping the caldera collapse), geochemical interpretation (guide for the interpretation of the faults), and geophysical surveys (guide for shaping the geological structures). The integrated geomodel^1^ intends to (i) synthetize as many of these data sources as possible to produce a coherent interpretation of the structures and formations, and (ii) combine them to give an interpretation of the geological system behaviour.

**Fig. 12:**
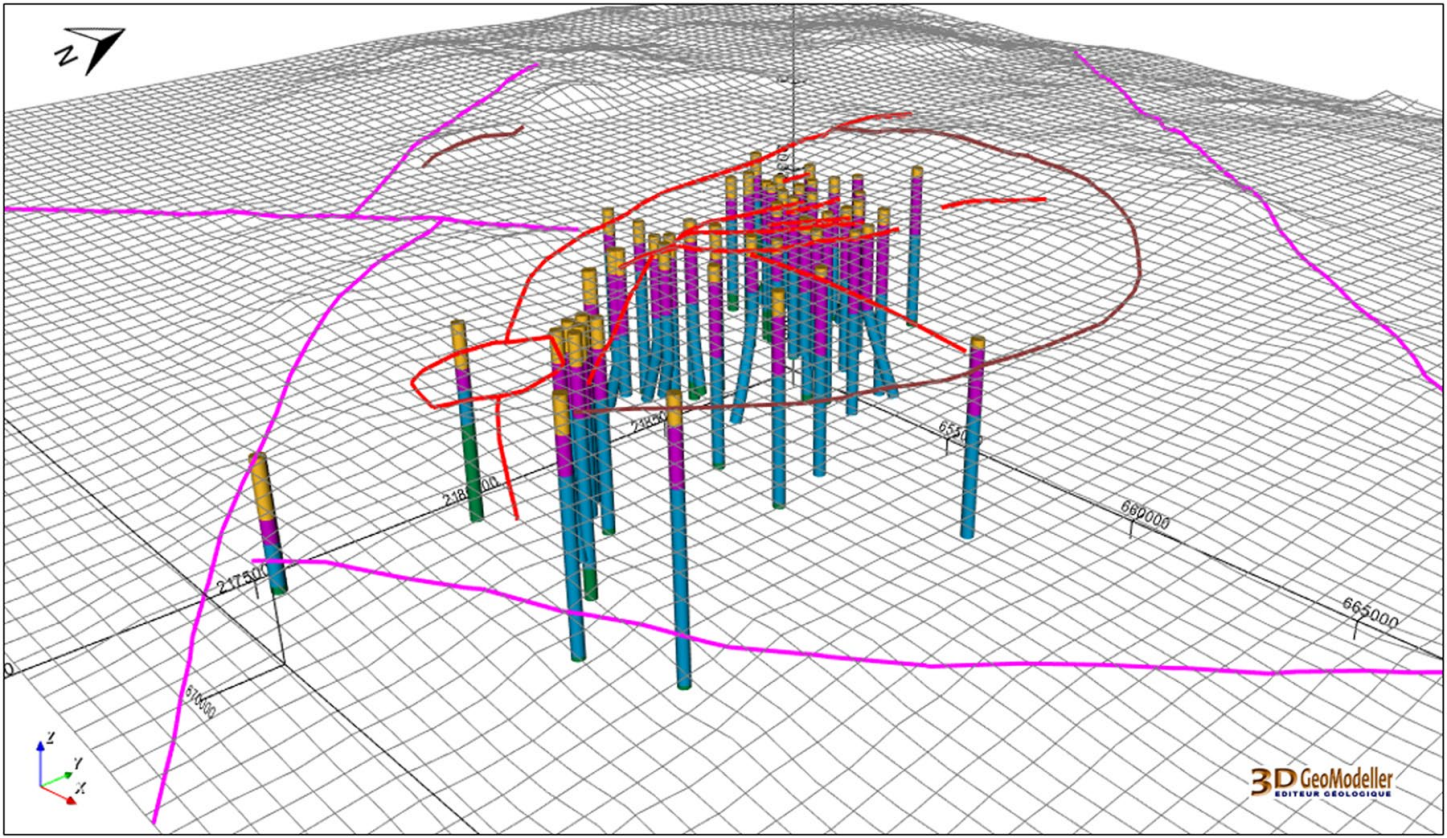
Fifty six wells are described according to the four groups 2018 version of the geological description presented in Fig. 5. DEM is displayed as a grid including the fault network traces (pink colour, see Fig. 13). The coordinate system is WGS84/UTM zone 14 N. View from SE.

The extent of the integrated geomodel (28 km × 22 km × 12 km, i.e. down to 7 km below sea level) was established primarily to span the horizontal extent of most of the geophysical surveys. The vertical extent of the integrated geomodel is the same than the regional and local ones. The 56 wells (Fig. 12) are described following the four groups of the geological description that is basement, pre-caldera rocks, rocks from the caldera, and post-caldera rocks (Figs. 4, 5).

The integrated geomodel is an evolution of the preliminary regional model and the updated local model. Several methods and data were used in an interdisciplinary approach to produce the integrated geomodel of Los Humeros, as presented in Table 1.

**Fig. 13:**
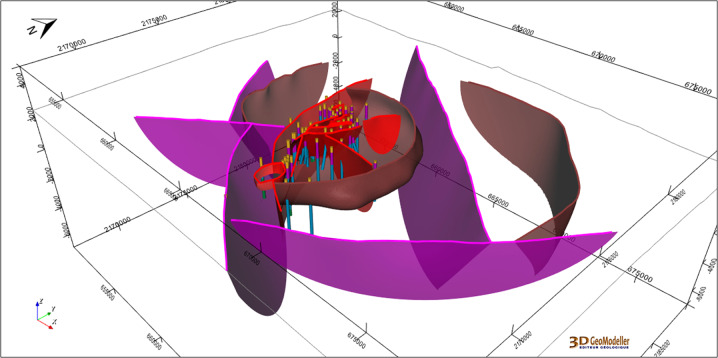
The fault network constructed in the Los Humeros integrated geomodel, along with the wells. Surfaces are visualized as semi-transparent to facilitate the interpretation of the figure. Purple: regional faults; brown: caldera structures; red: local faults. The coordinate system is WGS84/UTM zone 14 N.

One of the main updates of the integrated model regards regional faults. A new regional fault network was interpreted from the volcanic lineaments, which were considered as regional faults where permeability is favoured^24^. This set of regional faults is interconnected with the updated local fault network (Fig. 9). Figure 13 displays the whole fault network at the integration scale within the 3D geomodel.

**Fig. 14:**
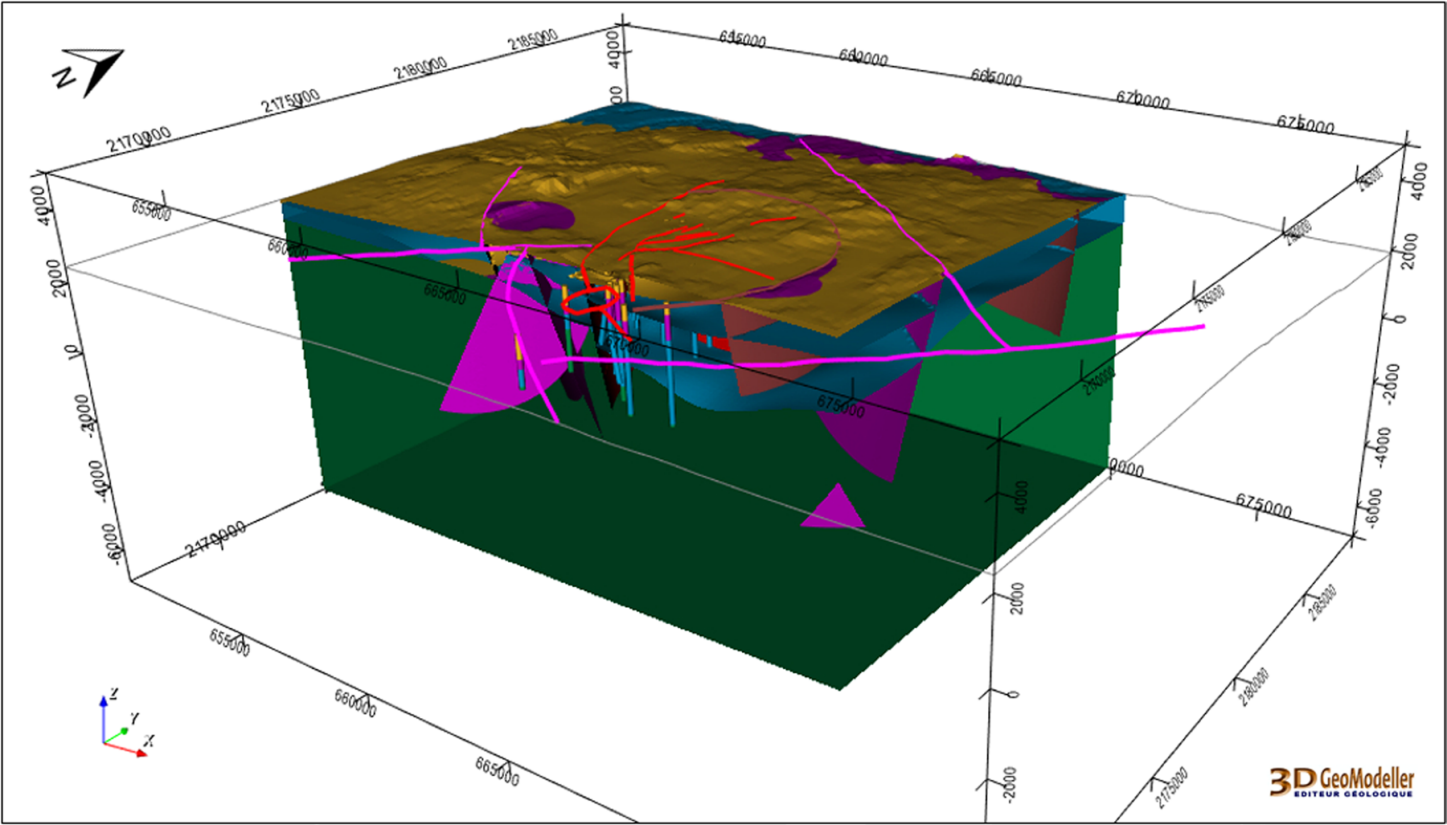
A clipped view of the Los Humeros integrated geomodel including wells, fault network, and the four geological groups: basement (green), pre-caldera rocks (blue), rocks from the caldera (purple), post-caldera rocks (brown). The coordinate system is WGS84/UTM zone 14 N.

The full integrated geomodel is presented in Fig. 14.

### Acoculco

The Acoculco geomodels were created at two scales to satisfy the needs of the exploration work (Fig. 2):

- A **local** scale focussing on the Acoculco area of the existing wells (i.e. EAC-1 and EAC-2)
- A **regional** scale corresponding the area covered by the fitting the geological map^22^.

The following sections present the input data and information, the geological description that was used, as well as the detailed integration process that was carried out to realise the geomodels.

#### Data and information

As with the Los Humeros, to perform the Acoculco 3D geomodels, the various datasets were imported into the GeoModeller package. Apart from the DEM of the study area, the initial geological datasets were: (a) the geological map of Acoculco^22^, constituted by a vector file with the main faults; (b) two interpreted geological cross-sections and (c) the litho-stratigraphic logs of the two deep boreholes drilled in the area^23^; (d) structural data from fieldwork carried out during GEMex. Afterwards, the interpretation derived from the geophysical dataset was embedded to constrain and refine the geomodel. Table 2 specifies the data used for the preliminary and updated geomodels, the additional data for the integrated geomodel, including the corresponding reference, as well as a description of the way those data were incorporated and used for the modelling.

**Table 2:** List and sources of datasets used for the construction of the Acoculco geomodels.

Geomodel version	Data	Use	Integration
Preliminary and updated regional geomodels	DEM (15 m horizontal resolution)^46^	To reproduce topographic surface	Imported as .tif
	Geological map^22^	To define the group of formations to be modelled - Compared with the resulting model (at surface)	Imported as .tif
	Geological cross-sections^32^	To define the geological structures at depth	imported as .tif
	Faults system network^24^	To define fractured zones	Imported as .shp file
	Boreholes Thermo-Litho-stratigraphy^23^	Constrain geological groups at depth	Imported as .csv
Local and integrated geomodels	3D gravity model^54^	Used to integrate geophysical dataset in the cross-plotting	Imported as .csv
	3D resistivity model^51^	Used to integrate geophysical dataset in the cross-plotting	Imported as .csv
	3D Thermal model^58^	Used to define the new intrusion shape, imported isotherm	Imported as .csv
	Depth of shallowest resistive basement from joint optimization of VES and TEM^51^	Used to check the depth of the vulcanites / limestones interface	Imported as .csv
	2D VES resistivity profile^51^	Used to check the depth of the vulcanites / limestones interface	Imported as .csv
	Distribution of the morpho-tectonic lineaments^49^	Checked to constrain the geological volcanic structures	Qualitative information
	Integrated geophysical model^55^	Imaging and validation of geological structures	Qualitative information
	Results from cross-plotting data integration^33^	Used as imported surfaces to check and refine the geomodel surfaces	Imported as .csv

The table indicates the purpose the dataset was used for and the way it was integrated in the geomodel. In the last column, “Qualitative information” means that the information was not directly imported but used for checking and evaluating the geomodel.

#### Geological description

The geological description adopted for the 3D Acoculco geomodel is the result of a synthesis driven by (a) the geothermal target (EGS); (b) the direct information available from the two drilled boreholes; (c) the geological map^22^ implemented by the results of the fieldwork survey, carried out in the GEMex project.

**Fig. 15:**
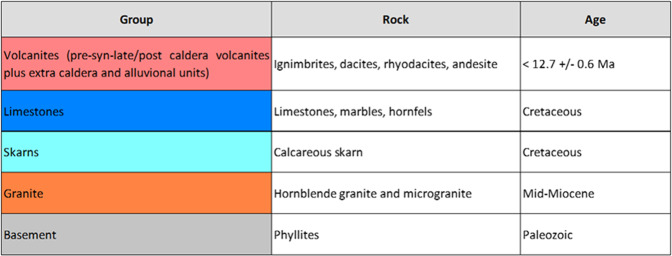
The geological description used for Acoculco^28^.

The regional geothermal gradient of the Acoculco area, as measured in the two wells, suggested useful temperatures for an EGS system deployment (i.e., more than 150 °C). These high temperatures were registered in the two boreholes at depths beyond the base of the volcanic rocks. For this primary reason, the construction of the geological description was devoted to the volcanic substratum, and thus all the volcanic rocks reported in the geological map^16,22^ were grouped into one single unit. Finally, taking into account the litho-stratigraphic information from the two wells^23^, the study of some outcrops, even in the reliefs to the east of Chignahuapan village and where bedrock is exposed^32^, the geological description displayed in Fig. 15 was set up and used to construct the Acoculco geomodels at regional and local scales.

#### Integration process

The geomodel of Acoculco was carried out using an interdisciplinary approach thanks to the contribution of the specialists who participated by providing not only datasets but also contributing in the periodic discussions to evaluate and improve the geomodel.

**Fig. 16:**
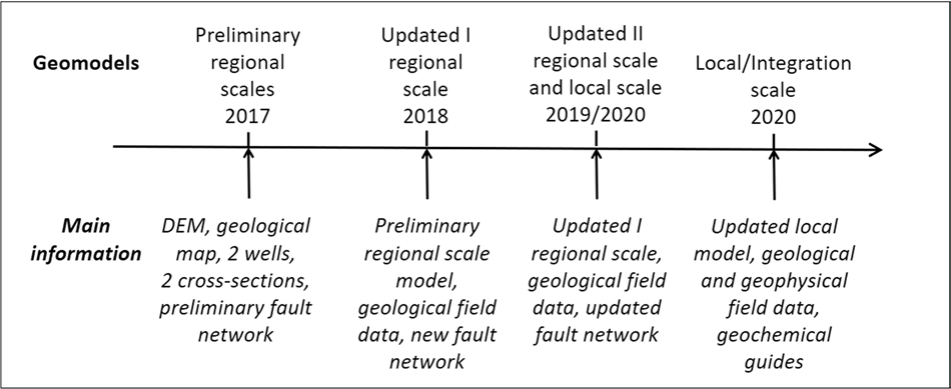
The versions of the Acoculco geomodels: preliminary^28^, updated version 1^29^, updated version 2, and integrated. The main information is listed for each of them.

The available versions of the geomodel of Acoculco are the result of several steps, which are schematically presented in Fig. 16.

#### Preliminary regional scale

**Fig. 17:**
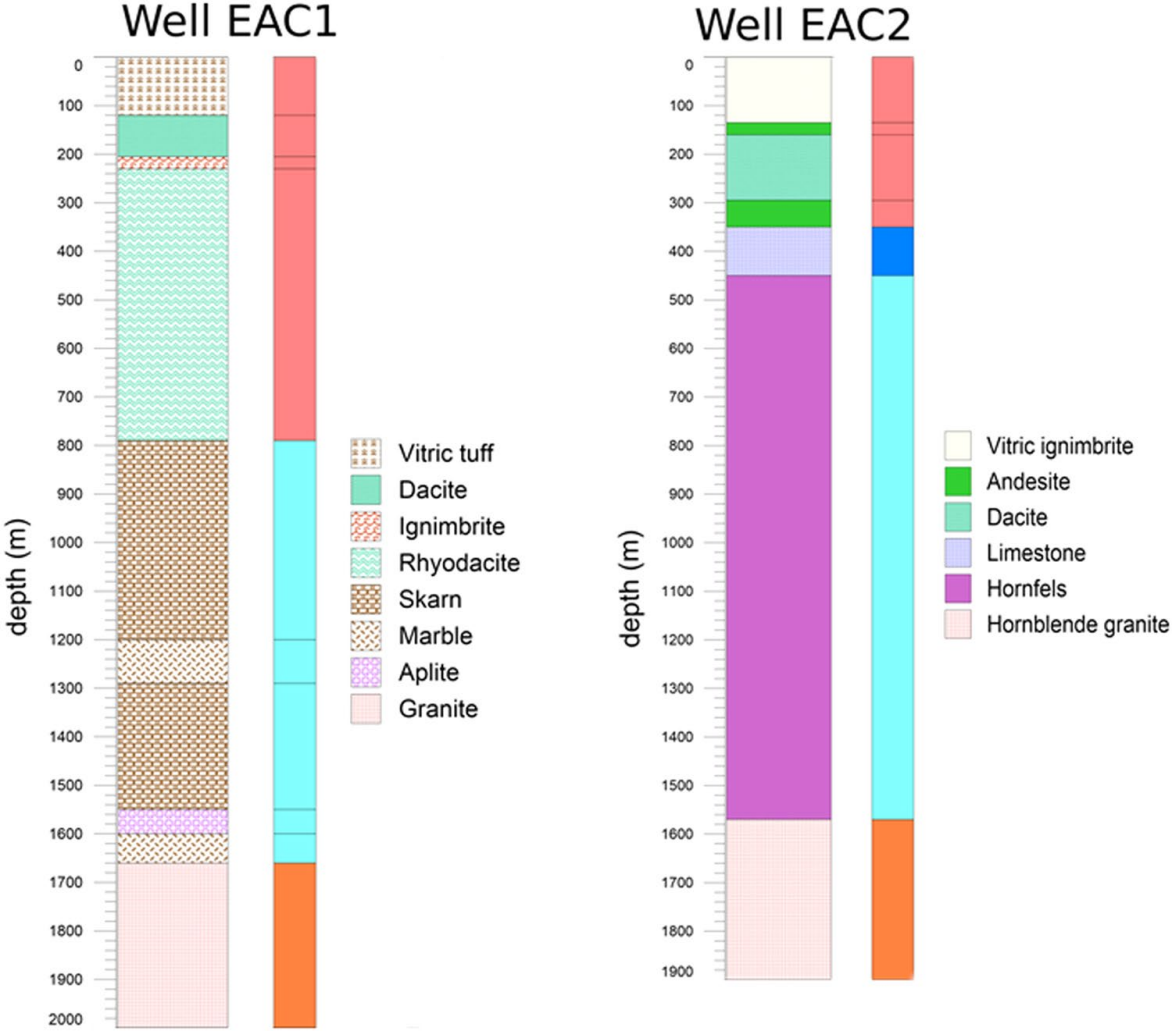
Geological description of wells EAC-1 and EAC-2^23^. For each of them, the slimmer column on the right side shows the geological units described in Fig. 15: granite (orange), skarns (light blue), limestones (blue), and volcanites (dark pink).

The preliminary model was carried out in the first year of the GEMex project and included mainly the existing data collected in the first months of the project^28^. Since only two boreholes (Fig. 17) drilled by CFE gave direct information on the underground, two interpreted geological cross-sections were prepared to constrain the model under the surface^28^. Apart the litho-stratigraphy of the two deep boreholes^23^, an European-Mexican joint group provided a first version of the faults network after initial field work.

The Acoculco area is intersected by NW-SE and NE-SW to ENE-WSW striking fault systems in mutual cross-cut relation, which suggests their contemporaneity. The faults belong to three different groups in terms of geometry and kinematics. The first group includes mainly NE-trending normal faults. The second group comprises NW-trending faults with a typical strike- to oblique-slip movement. The last group concerns secondary faults and part of the caldera-rim faults, which developed during the caldera collapse^28^.

The Digital Elevation Model (DEM) was provided by INEGI (Instituto Nacional de Estadística y Geografía) with a 15 m horizontal resolution.

Five rock groups were modelled (see Fig. 15). The basement, which is the planned geothermal target at Acoculco, was split into four groups while all the overlying volcanic rocks were combined into a single group. The basement includes, from bottom to top, phyllite, micaschist, limestone and skarn, intruded by a granitic rock^28^. 26 faults were modelled at the regional scale.

**Fig. 18:**
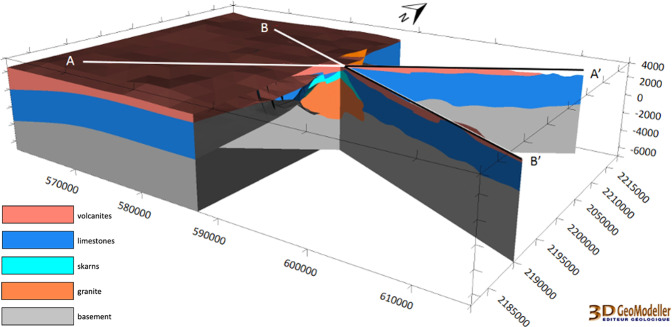
The Acoculco regional geomodel^28^. The coordinate system is WGS84/UTM zone 14 N.

The preliminary model was developed at regional scale and covered an area of 56 × 37 × 10.5 km; see Fig. 18.

#### Updated regional scale version 1

In 2018, an updated version of the regional scale Acoculco geomodel was developed^29^.

**Fig. 19:**
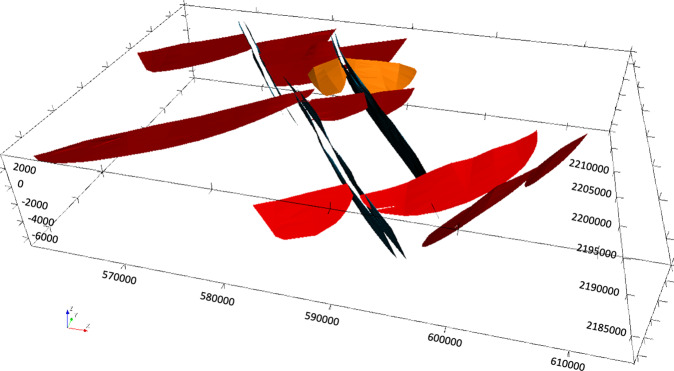
The updated faults system including the ‘Damage zones’, modelled as delimited by two sets of NNW-SSE striking parallel faults^29^. The coordinate system is WGS84/UTM zone 14 N.

This updated version was based on a revised fault network set up after a second round of fieldwork carried out by the joint European-Mexican group. The previous structural setting was modified mainly to take into account the observed NW-trending faults. Field observations identified the occurrence of individual fault segments, mainly associated in NW-trending brittle shear zones, thus defining two main brittle corridors, where permeability is expected to be increased somewhat relative to the surroundings. In order to highlight this new outcome, the updated map reports stripes described as ‘damage zones’ 500–600 m in width, (Fig. 19). Additional minor changes were related to the NE-SW oriented normal faults, in terms of their location or occurrences newly identified.

20 faults were modelled in this version.

**Fig. 20:**
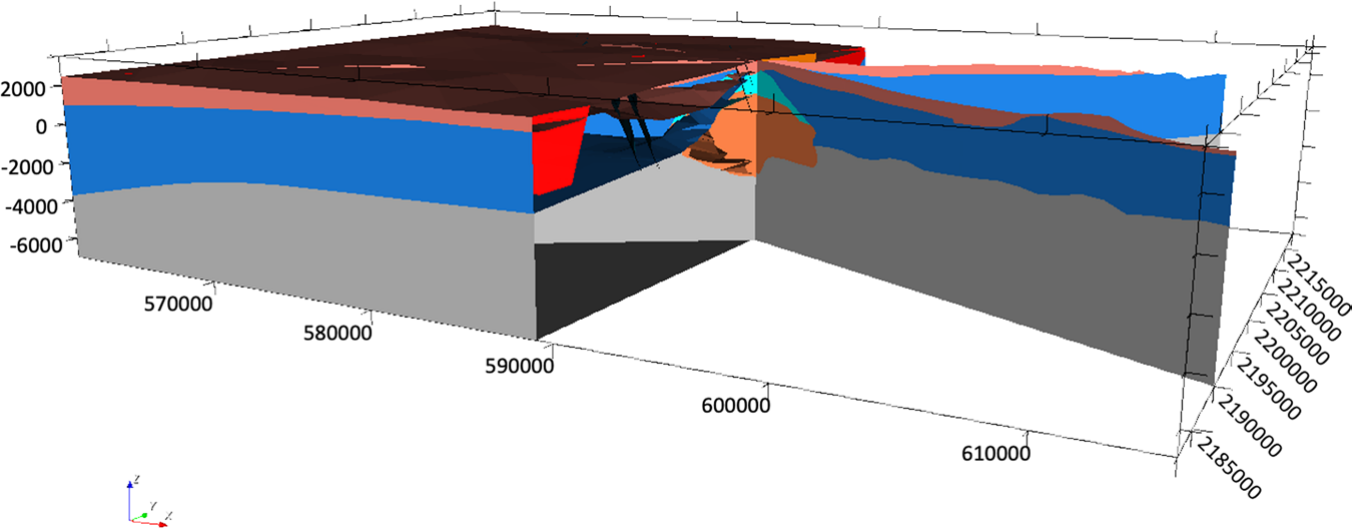
The updated regional 3D geomodel of Acoculco with the new faults and the ‘Damage zones’^29^. The coordinate system is WGS84/UTM zone 14 N.

The full updated geomodel is illustrated in Fig. 20.

#### Updated regional scale version 2

The second update of the regional scale geomodel was made after the final fieldwork of the project had concluded^1^. This fieldwork was focused mainly on selected key areas, collecting data specifically for the final understanding of the structures at regional scale. Moreover, a detailed geological survey was accomplished in the surroundings of the EAC-1 and EAC-2 boreholes, in that sector named as the local area.

**Fig. 21:**
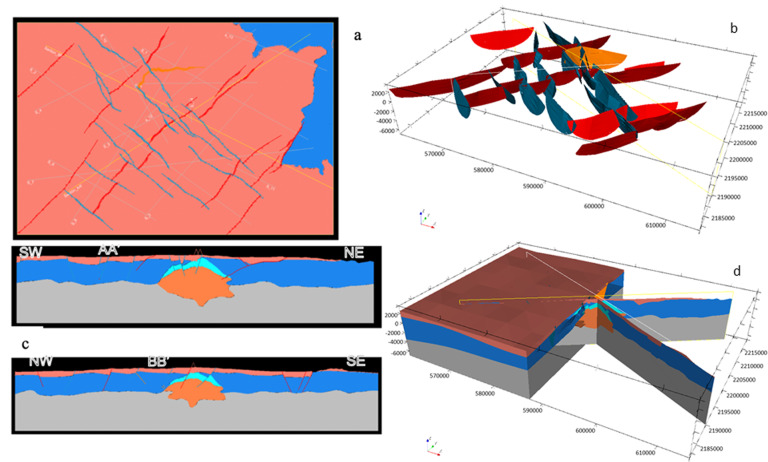
The second updated regional 3D geomodel of Acoculco with the new faults: (**a**) computed geological map; (**b**) 3D view of the faults network; (**c**) two computed geological cross-sections; (**d**) 3D sliced view of the geomodel.

Finally, the regional 3D geomodel of Acoculco was updated again based on the outcomes from the above mentioned fieldwork (Fig. 21). The principal difference with the previous version relates to the faults network. Field observations allowed to add a few new NE-SW striking normal faults and depict the NW-trending faults with more accuracy. 41 faults were modelled in this version.

As mentioned, the two deep boreholes EAC-1 and EAC-2 drilled by CFE did not produce any exploitable geothermal fluid. However, due to the high temperature registered at both bottom holes, the Acoculco site was dedicated to the preparatory studies for an EGS development in the GEMex project. Thus, geophysical surveys and stimulation modelling were focused nearby the two CFE boreholes.

#### Local scale

The new and more detailed information of the area around the EAC-1 and EAC-2 wells gave the possibility to prepare a detailed local model aimed at better characterising this area and to provide detailed geometries to be considered for the final design of the hydraulic stimulation test that was conducted by the Mexican consortium of the GEMex project.

**Fig. 22:**
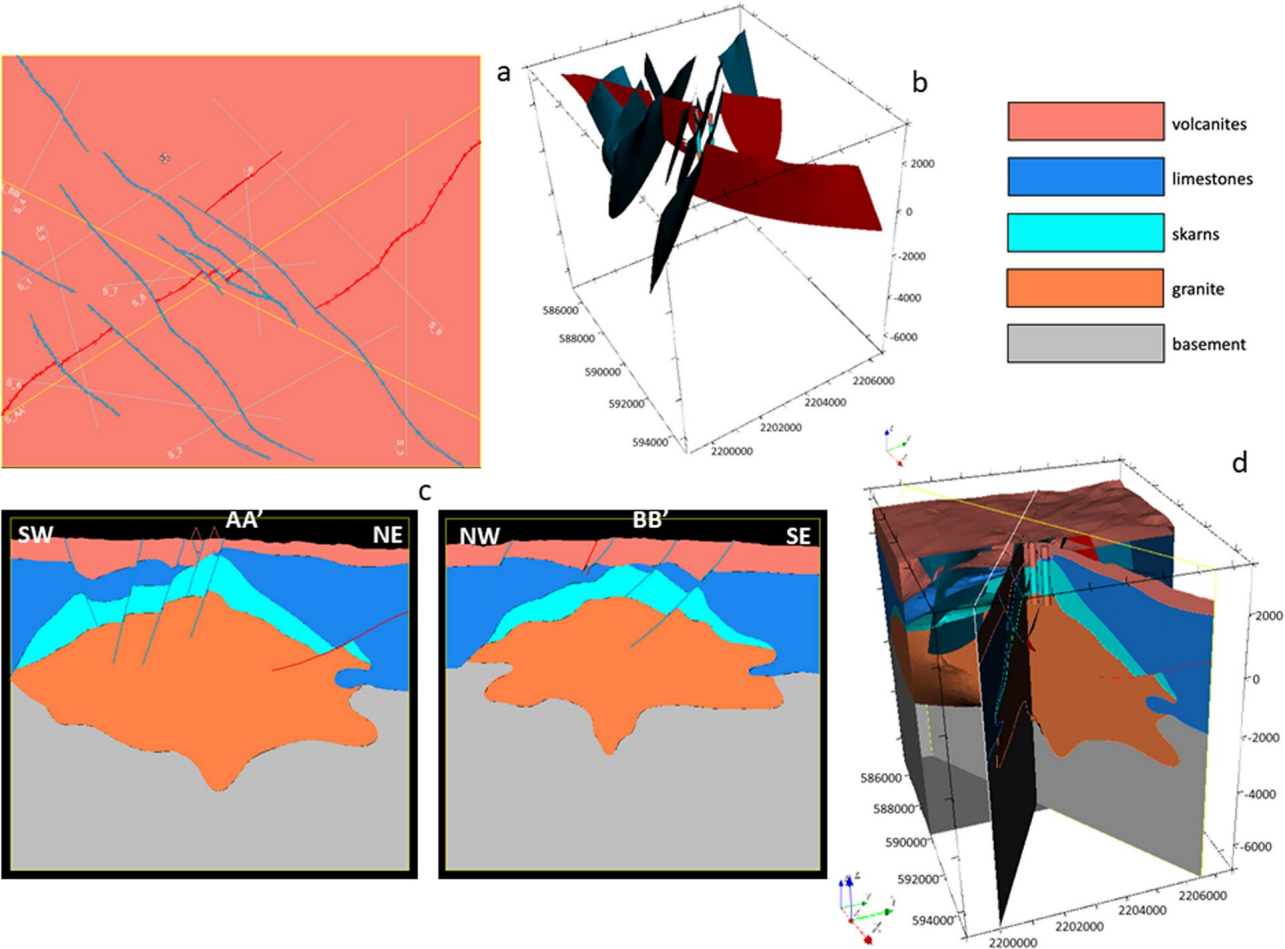
The updated local 3D geomodel of Acoculco with the detailed faults network: (**a**) computed geological map; (**b**) 3D view of the faults network; (**c**) two computed geological cross-sections; (**d**) 3D sliced view of the geomodel.

The local model^1^ is centred in the two boreholes and is 8.5 × 10.5 km horizontally spaced with a vertical dimension of 10.5 km in total. The model was built considering the results of the regional model, but it was refined with a more accurate and detailed faults network (Fig. 22). Small faults that do not appear in the regional model for scale reasons are included in this version. In this local model, the same five groups of geological units, shown in Fig. 15, were used (i.e., from the top: volcanites, limestones, skarns, granite, and basement). The two boreholes were included in the model again to constrain it with direct data. This version of the local model provided the base for the following integrated model with the geophysical outcomes.

The Digital Elevation Model (DEM) was provided by INEGI (Instituto Nacional de Estadística y Geografía) with a 15 m horizontal resolution.

16 faults were modelled at local scale.

#### Integration local scale

Various data were used to improve the accuracy of the Acoculco local geomodel. Most of them are the main outcomes of the geophysical surveys carried out in the local area during the GEMex project. The datasets considered for the integrated model are listed in Table 2.

An important contribution was provided by cross-plot and cluster analysis^33^ that allowed a quantitative integration of the different geophysical datasets. Cross-plot are used to interpret geophysical datasets and can suggest various correlation between variables with a certain interval of confidence^33^.

**Fig. 23:**
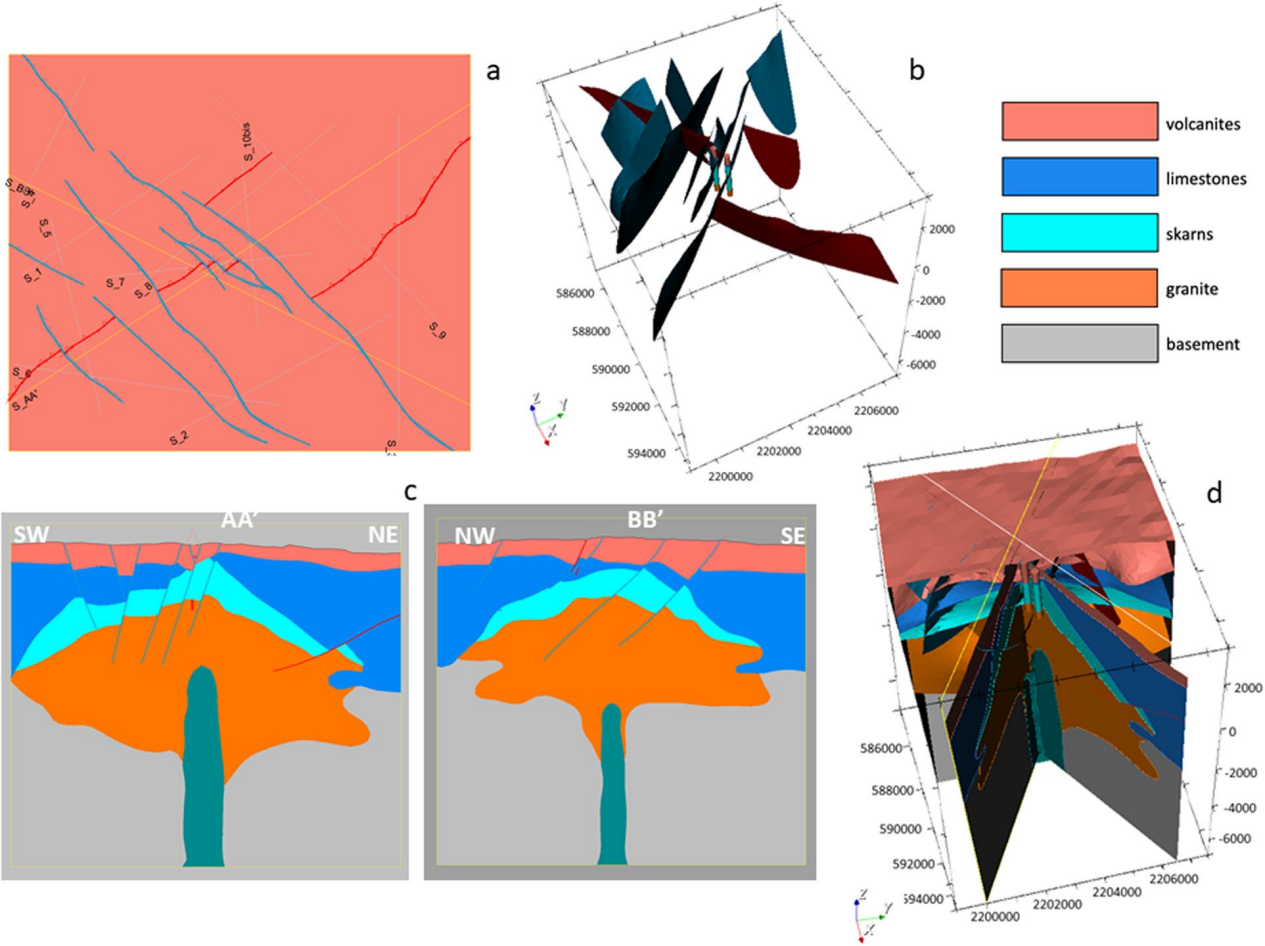
The integrated local 3D geomodel of Acoculco: (**a**) computed geological map; (**b**) 3D view of the faults network; (**c**) two computed geological cross-sections; (**d**) 3D sliced view of the geomodel. The coordinate system is WGS84/UTM zone 14 N.

The 3D integrated geological model at local scale^1^ is shown in Fig. 23. It includes: i) the update of the location of the base of the volcanites with the resistivity outcomes; ii) the update of the skarn volumes of rocks with the defined high resistivity – high density volumes of rock obtained by the cluster analysis; iii) the introduction of a new young magmatic intrusion gathered from the regional thermal model and iv) the confirmation of the fault added in the Alcaparrosa manifestations area.

16 faults were modelled at the integration scale.

## Data Records

The geomodels described in the previous section are recorded in the Zenodo repository under the Creative Commons Attribution 4.0 International (CC BY 4.0) license. They are listed in Tables 3, 4, respectively for Los Humeros and Acoculco. The geomodels come with a metadata sheet in pdf format which summarizes the technical parameters related to the model such as location, description and contact. Each geomodel is available in the form of the following files and formats:

- GeoModeller project format
- PDF3D format
- TSurf format
- VTK format

**Table 3:** List and repositories of the Los Humeros geomodels records presented in this study.

Los Humeros geomodels	Repository DOIs
Preliminary regional scale^59^	10.5281/zenodo.4604503
Preliminary local scale^60^	10.5281/zenodo.4607457
Updated local scale^61^	10.5281/zenodo.4607459
Integration scale^62^	10.5281/zenodo.4607460

**Table 4:** List and repositories of the Acoculco geomodels records presented in this study.

Acoculco geomodels	Repository DOIs
Preliminary regional scale^63^	10.5281/zenodo.4564252
Updated regional scale version 1^64^	10.5281/zenodo.4604452
Updated regional scale version 2^65^	10.5281/zenodo.4604467
Local scale^66^	10.5281/zenodo.4604470
Integration local scale^67^	10.5281/zenodo.4604479

## Technical Validation

The geomodels presented in this paper are deterministic constructions. Their quality depends on: i) the amount, quality, and diversity of the input data and information; ii) the interpolation approach; and iii) the collaborative/iterative process in which the models were developed.

It is important to mention that uncertainties cannot be computed when using the potential field interpolation method, except in some specific cases that are not met in this study^34^. Furthermore, the features of the input data were checked by the experts who produced them before the integration process described in this paper. Consequently, they are not discussed here. Please refer to the references in the second column of Tables 1, 2 for details. Instead, our validation discussion focuses on the way in which the data were integrated into the geomodels, and how the scientific acceptability of the results could be assessed.

Three approaches were followed to demonstrate the technical quality of our geomodels. The first one investigates the interpolation method, which has been applied in similar recent studies, yielding satisfactory results. The second relevant validation, available in the GeoModeller package, is the tool named *Drillhole properties* that reports the compliance of geomodels in regard to the geological contents of the wells. Finally, the last approach we adopted for the technical validation was grounded on the continuous check among experts, in a collaborative and interdisciplinary way where consensus was foreseen as much as possible.

### Robustness of the interpolation method

The input data were interpolated using a scalar field method to generate surfaces and volumes of the geological objects^25,26^ (see the “Geomodelling” part of the “Methods” section above). The robustness and efficiency of this co-kriging method has been proven by models constructed in various geological contexts for several applications^35–44^.

In particular, the same potential field interpolation method embedded in the same software (GeoModeller) was used to successfully develop a geological model of a complex Alpine area^42^. The authors discussed from a technical perspective the interpolation applied to the data of their zone. Even if this discussion is illustrated by their own data, most of the outcomes are relevant for any study using the potential field interpolation method. Thus, rather than repeat here the main conclusions of the authors to sustain the reliability and robustness of the interpolation method used by the GeoModeller, we kindly refer interested readers to that work.

### Quality control: Conformity between the geomodels and the geological description of the wells

The most objective data used to build the geomodels are the geological description of the lithological column cut by the wells. This section presents how the accuracy of the geomodels was checked in regard to the geological description of the wells.

It is important to remark that the interpolation method used to construct the geomodels computes 3D scalar fields^25,26^. This method is very sensitive to inconsistencies that may occur in the data. For instance, two close wells with a different geological description will generate instabilities in the interpolation. This property is useful to detect and either (i) to fix such inconsistencies in the geological description of the wells and sometimes even a wrong location of the well, or (ii) to interpret geological features, e.g. a fault, able to explain the discrepancy between two close wells. For this reason, checking and evaluating the behaviour of the interpolation with regard to the description of the wells is a crucial part of the modelling process that was applied to the geomodels of Los Humeros. The presence of only two boreholes with scarce logs in Acoculco didn’t allow us to perform a similar quality control in this area.

Another important issue in handling wells regards inequalities. The description of the well is sometimes not enough comprehensive to be used in an unequivocal manner. As an example, the bottom of a well generally does not mark a geological interface, i.e. the limit between two geological formations. It implies that a bottom of the well cannot be used as a point for the interpolation. However, it indicates that the next geological interface is located deeper that the bottom of the well. Consequently, it is necessary to check if the lower geological interface is indeed interpolated below the bottom of that specific well. If not, a 3D point associated to the geological interface - an equality constraint - has to be added below the bottom of the well to honour it. This procedure was applied during the development of the geomodels presented in this study.

The goal of the quality control is not to check how the geomodel can predict geology, for instance by withholding well data and compare the model with the actual well description. As stated in a previous study: “… no data were explicitly withheld to provide independent evaluation data; keeping any observations aside would inevitably have had an adverse impact on the final model.”^42^. The geomodels quality control is a quantified approach based on the comparison of the computed well contents, i.e. the well extracted from the geomodel, and the geological description of the same well that was used as input data. approach allows us to check that geological inconsistencies are fixed in the description of the wells, and inequalities are properly tackled.

**Fig. 24:**
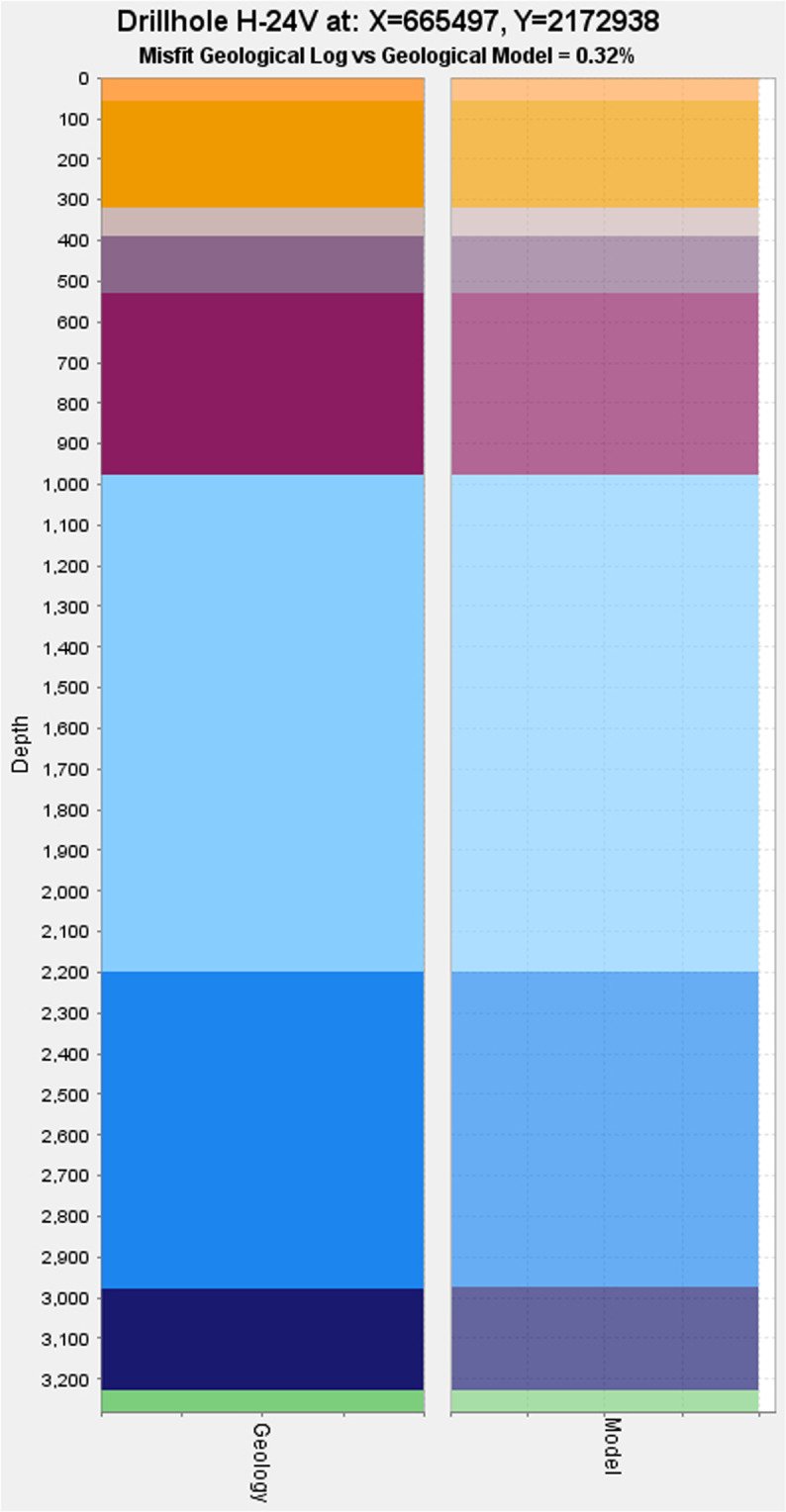
Comparison of the well H-24V geological description used as input data (left side) and the same well extracted from the Los Humeros updated local scale geomodel (right side). The misfit between them is 0.32%.

The misfit between the original well and the modelled one is quantified as a percentage. A lower percentage indicates a better fit between the actual well description and the modelled one. The quality control is achieved via the GeoModeller tool named *Drillhole properties*. As an example, Fig. 24 shows how well H-24V, one of the 56 wells used for the Los Humeros updated local scale, is taken into account by the geomodel.

**Fig. 25:**
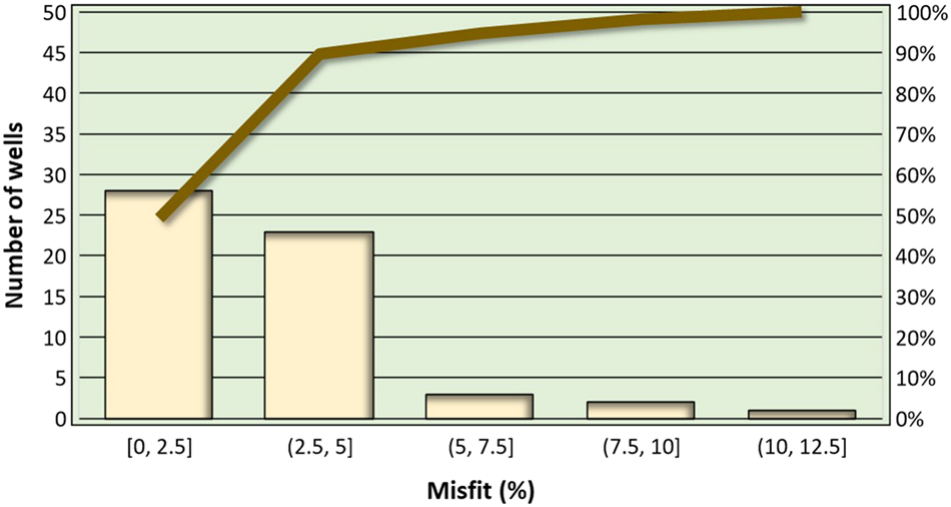
Los Humeros updated local scale geomodel. Histogram of the misfits between the geological description and modelled geology in the wells (yellow). The Pareto line (brown) shows that 90% of the wells have a misfit lower than 5%.

The computation presented in Fig. 24 was applied to all the wells used for the updated local and integration scales of the Los Humeros geomodels. For the updated local scale, results are displayed in Fig. 25. It shows that 28 wells (50% of the total), represented by the first bar of the histogram, present a misfit lower than 2.5% (i.e., between 0 and 2.5%), and 50 wells (90% of the total), represented by the first and second bars of the histogram, present a misfit lower than 5% (i.e., between 0 and 5%).

**Fig. 26:**
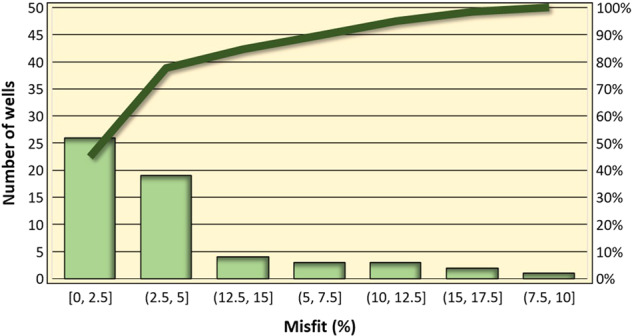
Los Humeros integration scale geomodel. Histogram of the misfits between wells geological description and modelled geology (light green). The Pareto line (green) shows that almost 80% of the wells have a misfit lower than 5%.

The misfits of the 56 wells used for the Los Humeros geomodel at the integration scale are a little higher than at the update local scale, as shown in Fig. 26. In this case, 26 wells (44% of the total) included in the first histogram present a misfit lower than 2.5%, and 44 wells (78.5% of the total) present a misfit lower than 5%, as shown by the first and second histograms.

In complement of this quantitative control that focus on the wells location and description, the reliability of the geomodels benefits from the expert evaluation of whether the model is plausible elsewhere, as described in the next section.

### A collaborative and interdisciplinary way of working to maximize knowledge reliability

One of the main outcomes of constructing a geological model is a coherent interpretation in three dimensions. Merging the data in the same 3D space enabled possible inconsistencies to be checked and corrected. Moreover, being able to visualize and to easily interact with the modelled geological objects is a powerful way of sharing a common view of the geology among a group of people. The modelling process can thus be used as a collaborative platform for exchange and debate, and for agreeing upon the geological interpretation.

One of the major issues, when constructing a geomodel, is that input data are often sparse and irregularly disseminated. They can be dense in some places corresponding to acquisitions location, such as along a well, but all the rest of the space to be investigated remains blind in terms of data. For this reason, it is necessary to be able to assess and verify the model plausibility away from the input data locations. Usually, a reliable geomodel cannot be properly constructed by a single person, as such work not only relies on the merging of data, but also on integrating multiple knowledge sources and interpretations.

The Los Humeros and Acoculco geomodels were thus constructed as collaborative efforts. Two teams, one dedicated to Los Humeros and the other to Acoculco, gathered scientists from Europe and Mexico. All data to be considered were initially discussed among the specialists responsible for the construction of the geomodels (Figs. 1 and 2). Then, a loop was established through three main steps: (i) gathering existing data & knowledge, (ii) modelling, (iii) evaluating and validating the model. The models were discussed at the team level. Each step of integration was done by means of dedicated meetings, in order to maintain the high-level of cooperation and correctness of the geological interpretation. Possible issues were thus identified and debated until an agreement was reached by the experts. In some cases, issues were suspended up to have new data, and then the revised data and knowledge was input in a new loop until the models were fully evaluated by the partners.

Geologists, volcanologists, geophysicists and modellers contributed to the development of the geomodels. Considering the international mix of scientists in the teams, a critical issue of the collaboration resided in communication. In order to render the collaborative process as efficient as possible, tele-workshops dedicated to working sessions based on interactive exchanges were organized on a regular basis. The work relied on media such as cross sections complementary to the ones described in the “Methods” section above. They were used to check, constrain and evaluate the geomodels. In Los Humeros, 11 complementary cross sections were used at the regional scale and one at the local scale. In Acoculco, 13 complementary cross sections were used at the regional scale and 10 at the local scale. Furthermore, protocols such as the use of 3D PDF files were set up for exchanging the 3D models, which allowed the team members to visualize and check the steps of the construction.

## Usage Notes

The geomodels are shared on the Zenodo repository under the Creative Commons Attribution 4.0 International (CC BY 4.0) license. They are in the original GeoModeller format, PDF 3D,TSurf, and VTK formats, readable using the tools listed in Table 5.

**Table 5:** Geomodels file formats available on the Zenodo repository and software to read them.

File format	Software
GeoModeller	GeoModeller, https://www.geomodeller.com
3D PDF	Standard Adobe pdf reader, https://get.adobe.com/fr/reader/
TSurf	GocadTSurfaceReader, https://www.opengeosys.org/docs/tools/fileio/gocadtsurfacereader/
VTK	Paraview, https://www.paraview.org/download/

## Data Availability

The geomodels presented in this paper were constructed using the GeoModeller commercial package, which was developed by BRGM, the French Geological Survey, with the support of Intrepid Geophysics. This software is based on original methodologies for geological and geophysical modelling^25,26,45^. More information at: https://www.geomodeller.com. The following versions of GeoModeller were used to achieve the geomodels presented in this study. *Los Humeros:* GeoModeller Version: 4.0.7 Build Date: May 22 2019 Build Number: 27eee3dc31ba *Acoculco:* GeoModeller Version: 4.0.8 Build Date: Aug 04 2020 Build Number: d783d7694b8 The default parameters of GeoModeller were used for the interpolation of all the geomodel versions presented in this paper.
